# SimC7 Is a Novel NAD(P)H-Dependent Ketoreductase Essential for the Antibiotic Activity of the DNA Gyrase Inhibitor Simocyclinone

**DOI:** 10.1016/j.jmb.2015.03.019

**Published:** 2015-06-19

**Authors:** Martin Schäfer, Tung B.K. Le, Stephen J. Hearnshaw, Anthony Maxwell, Gregory L. Challis, Barrie Wilkinson, Mark J. Buttner

**Affiliations:** 1 Department of Molecular Microbiology, John Innes Centre, Norwich Research Park, Norwich NR4 7UH, United Kingdom; 2 Department of Biological Chemistry, John Innes Centre, Norwich Research Park, Norwich NR4 7UH, United Kingdom; 3 Department of Chemistry, University of Warwick, Coventry CV4 7AL, United Kingdom

**Keywords:** BBSRC, Biotechnology and Biological Sciences Research Council, SD8, simocyclinone D8, SDR, short-chain dehydrogenase/reductase, MIC, minimum inhibitory concentration, HMBC, heteronuclear multiple bond correlation, LC-MS, liquid chromatography mass spectrometry, PAC, phage artificial chromosome, simocyclinones, DNA gyrase, antibiotics, short-chain dehydrogenase/reductase (SDR) superfamily, ketoreductase

## Abstract

Simocyclinone D8 (SD8) is a potent DNA gyrase inhibitor produced by *Streptomyces antibioticus* Tü6040. The simocyclinone (*sim*) biosynthetic gene cluster has been sequenced and a hypothetical biosynthetic pathway has been proposed. The tetraene linker in SD8 was suggested to be the product of a modular type I polyketide synthase working *in trans* with two monofunctional enzymes. One of these monofunctional enzymes, SimC7, was proposed to supply a dehydratase activity missing from two modules of the polyketide synthase. In this study, we report the function of SimC7. We isolated the entire ~ 72-kb *sim* cluster on a single phage artificial chromosome clone and produced simocyclinone heterologously in a *Streptomyces coelicolor* strain engineered for improved antibiotic production. Deletion of *simC7* resulted in the production of a novel simocyclinone, 7-oxo-SD8, which unexpectedly carried a normal tetraene linker but was altered in the angucyclinone moiety. We demonstrate that SimC7 is an NAD(P)H-dependent ketoreductase that catalyzes the conversion of 7-oxo-SD8 into SD8. 7-oxo-SD8 was essentially inactive as a DNA gyrase inhibitor, and the reduction of the keto group by SimC7 was shown to be crucial for high-affinity binding to the enzyme. Thus, SimC7 is an angucyclinone ketoreductase that is essential for the biological activity of simocyclinone.

## Highlights

• The ~ 75-kb simocyclinone biosynthetic cluster was expressed in a heterologous system.
• SimC7 is a novel NAD(P)H-dependent ketoreductase.
• SimC7 function is essential for the antibiotic activity of the DNA gyrase inhibitor simocyclinone.

## Graphical abstract

**Figure:**
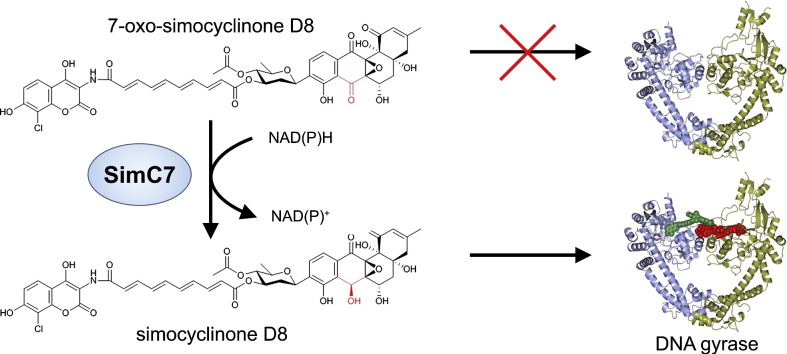

## Introduction

**Fig. 1:**
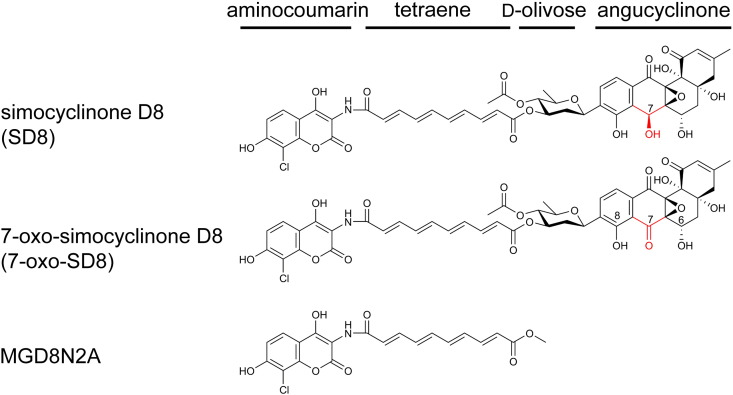
Chemical structures of simocyclinone SD8, the major product of *S. antibioticus* Tü6040; 7-oxo-SD8, the major product of the *simC7* mutant; and MGD8N2A, a semi-synthetic analog lacking the angucyclinone polyketide moiety (generated by chemical hydrolysis of SD8). The absolute stereochemistry of SD8 was determined from co-crystals with SimR (PDB accession number 2Y30) [14] and *E. coli* DNA gyrase (PDB accession number 4CKL) [8]. The C-6, C-7, and C-8 positions discussed in the text are numbered, and the structural difference between SD8 and 7-oxo-SD8 is highlighted in red.

Simocyclinone D8 (SD8) is a natural hybrid antibiotic made by *Streptomyces antibioticus* Tü6040. It consists of a chlorinated aminocoumarin connected to an angucyclic polyketide via a tetraene diester linker and a d-olivose deoxysugar [1] (Fig. 1). SD8 is a potent inhibitor of supercoiling by DNA gyrase, an enzyme that is essential in bacteria but is absent from humans, making it an excellent target for antimicrobial drugs, as illustrated by the clinically important fluoroquinolones [2–4].

All known aminocoumarin antibiotics target bacterial DNA gyrase, but their modes of action differ. The three classical *Streptomyces* aminocoumarin antibiotics—novobiocin, clorobiocin, and coumermycin A1—bind to the GyrB subunit of DNA gyrase and inhibit ATPase activity [5]. In contrast, SD8 binds to the GyrA subunit of DNA gyrase and prevents binding to DNA [6]. SD8 is a bifunctional antibiotic with the aminocoumarin and angucyclic polyketide moieties at either end of the molecule binding to two separate pockets on the DNA-binding interface of GyrA [7,8]. The interaction with GyrA shows positive cooperativity, with the binding of one end of SD8 to its pocket promoting binding of the other end to its pocket. Based on these observations, it has been proposed that binding to GyrA is initiated by the high-affinity angucyclic polyketide, followed by improved binding of the low-affinity aminocoumarin [9]. Because of the bifunctional nature of simocyclinone, removal of either the aminocoumarin or the angucyclic polyketide from the antibiotic reduces its potency as a DNA gyrase inhibitor by about 100-fold compared to SD8, which inhibits DNA supercoiling at sub-micromolar concentrations (IC_50_ ~ 0.1–0.6 μM) [7,9].

Based on the intermediates detectable in cultures of *S. antibioticus* Tü6040, it seems that the biosynthesis of SD8 starts with assembly of the angucyclic polyketide, followed by the attachment of the d-olivose deoxysugar, then the tetraene linker, and finally the aminocoumarin (i.e., assembled from right to left as drawn in Fig. 1) [10]. Accordingly, simocyclinone intermediates are classified into four groups (A–D), with A-group intermediates having only the angucyclic polyketide moiety, B-group intermediates having the angucyclic polyketide linked to the d-olivose deoxysugar, and so on [10].

The 49-gene simocyclinone (*sim*) biosynthetic cluster of *S. antibioticus* Tü6040 has been sequenced and a hypothetical biosynthetic pathway has been proposed [11,12]. Although two *sim* cluster transcriptional regulators have been studied [13–16], to date, only one biosynthetic enzyme has been characterized. SimD5 (SimL) catalyzes the presumed last step in the pathway, functioning as an amide-bond-forming ligase that attaches the aminocoumarin to the tetraene linker [17–19].

Trefzer *et al.* proposed that the tetraene linker in SD8 would be the product of a modular type I polyketide synthase, SimC1ABC, working *in trans* with two monofunctional enzymes, SimC6 and SimC7 [11]. One of these monofunctional enzymes, SimC7, a member of the short-chain dehydrogenase/reductase (SDR) superfamily, was proposed to supply the dehydratase activity missing from two modules of the polyketide synthase [11]. Here, we determine the true function of SimC7 experimentally and show that it is an angucyclinone ketoreductase that is essential for the antibiotic activity of simocyclinone.

## Results

### Heterologous expression of simocyclinones

Although directed mutations have been successfully created in the SD8 producer, *S. antibioticus* Tü6040 [11,12], this strain is particularly challenging to manipulate genetically. As a consequence, we instead chose to clone the *sim* gene cluster and analyze it in a heterologous system. The whole *sim* cluster was isolated on a single phage artificial chromosome (PAC) clone (PAC-12I) after PCR screening of a genomic library generated in *Escherichia coli* according to a recently developed protocol for working with large gene clusters [20]. Sequencing of the 95-kb insert in PAC-12I revealed the presence of the whole minimal *sim* gene cluster, consisting of 49 genes covering an ~ 72-kb region (Fig. S1), flanked by genomic regions of ~ 19 kb and 4 kb on either side.

We conjugated PAC-12I from the methylation-deficient *E. coli* strain ET12567 into the heterologous host *Streptomyces coelicolor* M1152. This strain has been engineered for improved heterologous production and analysis of secondary metabolites by deletion of endogenous secondary metabolic clusters, resulting in a strain without antimicrobial activity and a much simplified extracellular metabolite profile for high-performance liquid chromatography (HPLC) and mass spectrometry analyses [21]. In addition, it carries a point mutation in the *rpoB* gene (encoding the RNA polymerase β-subunit) that causes a pleiotropic increase in the level of expression of secondary metabolic clusters [21]. PAC-12I strongly impaired the growth and sporulation of M1152. In an attempt to overcome this toxicity, we took advantage of a plasmid (pIJ10480 [13]) that expresses the *simEx1* simocyclinone efflux pump gene from the strong constitutive promoter ermEp*. This plasmid was previously shown to increase the minimum inhibitory concentration (MIC) of SD8 for *Streptomyces lividans* from 2 μg/mL to 65 μg/mL [13]. To improve heterologous production of simocyclinones, we introduced pIJ10480 into M1152, resulting in strain *S. coelicolor* M1152ex1. Overexpression of *simEx1* increased the MIC of SD8 for M1152 from 1 μg/mL to 100 μg/mL.

We introduced PAC-12I and an empty vector control into the optimized heterologous host M1152ex1 by conjugation. Overexpression of the *simEx1* simocyclinone efflux pump gene reduced the toxicity of PAC-12I and M1152ex1 was used for all further work. Strains were grown in a defined production medium consisting of a basic nutrient medium with l-glutamine (5.84 g/L) and glycerol (20 mL/L) as nitrogen and carbon sources, respectively [10]. SD8 production in the heterologous host (24–56 mg/L) was 2- to 5-fold higher than that in the natural producer *S. antibioticus* Tü6040 (9–14 mg/L). No simocyclinones were detectable from M1152ex1 control strains lacking the *sim* gene cluster.

### Structural analysis of a novel simocyclinone intermediate

**Fig. 2:**
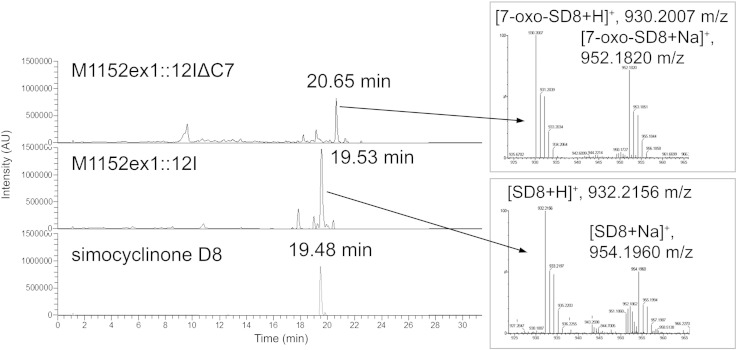
LC-MS-UV analysis of simocyclinones produced by expression of the *sim* gene cluster in the heterologous host *S. coelicolor* M1152ex1. Chromatograms (200–700 nm) of culture extracts from clones carrying the *simC7* mutant cluster on PAC 12IΔ*simC7* (top) and the complete *sim* cluster on PAC 12I (middle), alongside an isolated standard of SD8 (bottom), are shown. Molecular weights were confirmed by high-resolution mass spectrometry (parent peaks are indicated; full tandem mass spectrometry data can be found in Fig. S3). Cultures were grown for 6 days at 30 °C in production medium.

In order to determine the role of SimC7 in simocyclinone biosynthesis, we created an in-frame deletion in *simC7* in PAC-12I using λ-Red PCR targeting, and we introduced the resulting PAC clone (PAC-12IΔC7) into M1152ex1. When fermentation extracts of the resulting *simC7* mutant strain were analyzed by HPLC, the major biosynthetic product had a later retention time than that of SD8 (Fig. 2). The UV–vis spectrum of the new compound was similar to that of SD8 but with reduced absorbance at 275 nm and a slight shift in the absorbance maxima to 245 nm and 361 nm (Fig. S2). Unexpectedly, the major product of the *simC7* mutant had a mass 2 Da lighter than SD8. All of the A-, B-, and C-group simocyclinone intermediates made by the *simC7* mutant were also 2 Da lighter than the equivalent molecules made by the wild-type *sim* cluster, suggesting that the 2-Da difference lies in the angucyclic polyketide and not in the tetraene linker. Based on this observation, we predicted that one of the five hydroxyl groups present in the angucyclic polyketide moiety of SD8 remained as a carbonyl group in this molecule and that SimC7 acts as a ketoreductase. To test this hypothesis, we purified the new simocyclinone intermediate from 4 L culture and determined its structure (63 mg isolated yield).

**Fig. 3:**
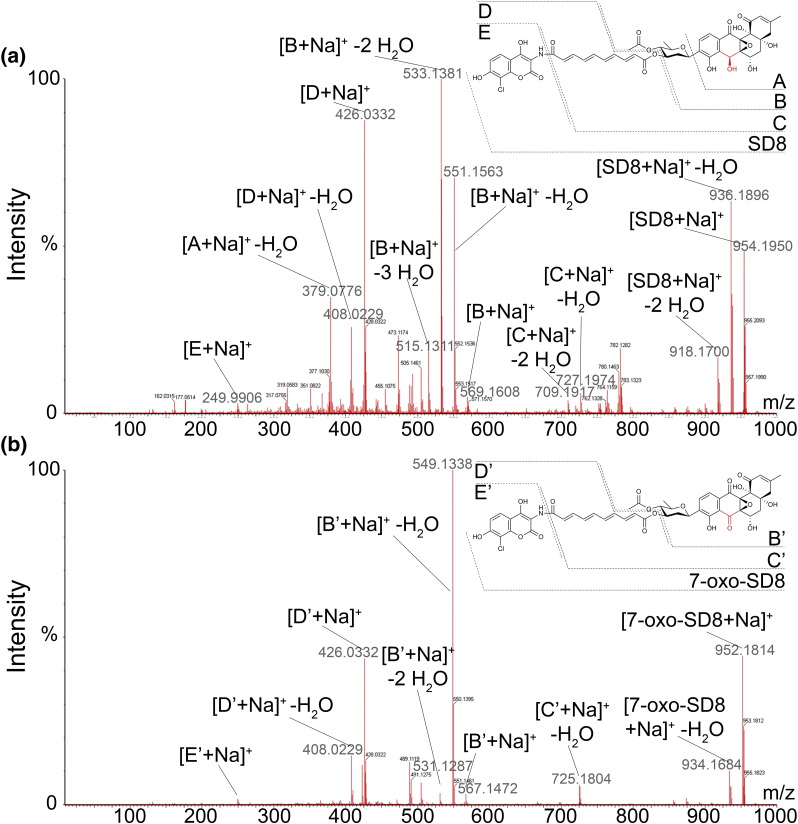
High-resolution tandem mass spectra for the sodium adducts of (a) SD8 and (b) 7-oxo-SD8. Selected fragments are highlighted, consistent with the mass difference of 2 Da being located in the angucyclic polyketide moiety.

The molecular formula of the new compound was determined using high-resolution mass spectrometry, which confirmed that it was missing two hydrogen atoms {*m*/*z* (electrospray ionization) for C_46_H_40_ClNO_18_: calculated, 930.2007; observed, 930.2009; Δ = 0.3 ppm [M + H]^+^} compared to SD8 {*m*/*z* (electrospray ionization) for C_46_H_42_ClNO_18_: calculated, 932.2163; observed, 932.2156; Δ = − 0.8 ppm [M + H]^+^} (Fig. 2 and Fig. S3). Tandem mass spectrometry analysis revealed similar fragmentation patterns for the new compound and SD8, with all of the ion fragments containing the angucyclic polyketide moiety having *m*/*z* ratios reduced by 2 Da compared to SD8. Conversely, all ion fragments from the new compound lacking the angucyclic polyketide moiety had identical masses with those derived from SD8 (Fig. 3).

**Fig. 4:**
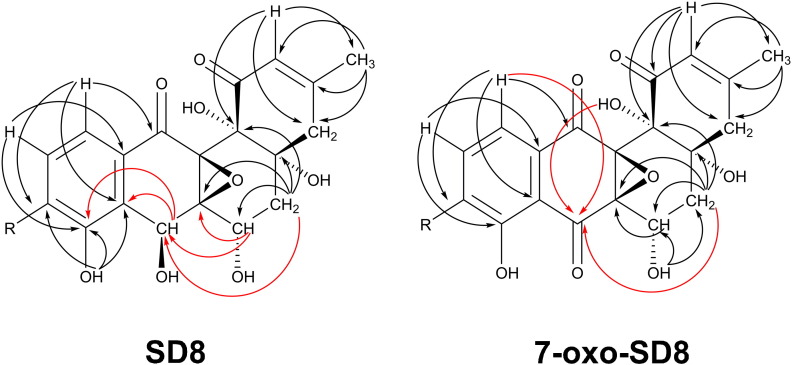
Selected HMBC correlations for the angucyclic polyketide moiety of SD8 and of the new simocyclinone intermediate 7-oxo-SD8. Key correlations for the C-7 position are depicted in red.

**Fig. 5:**
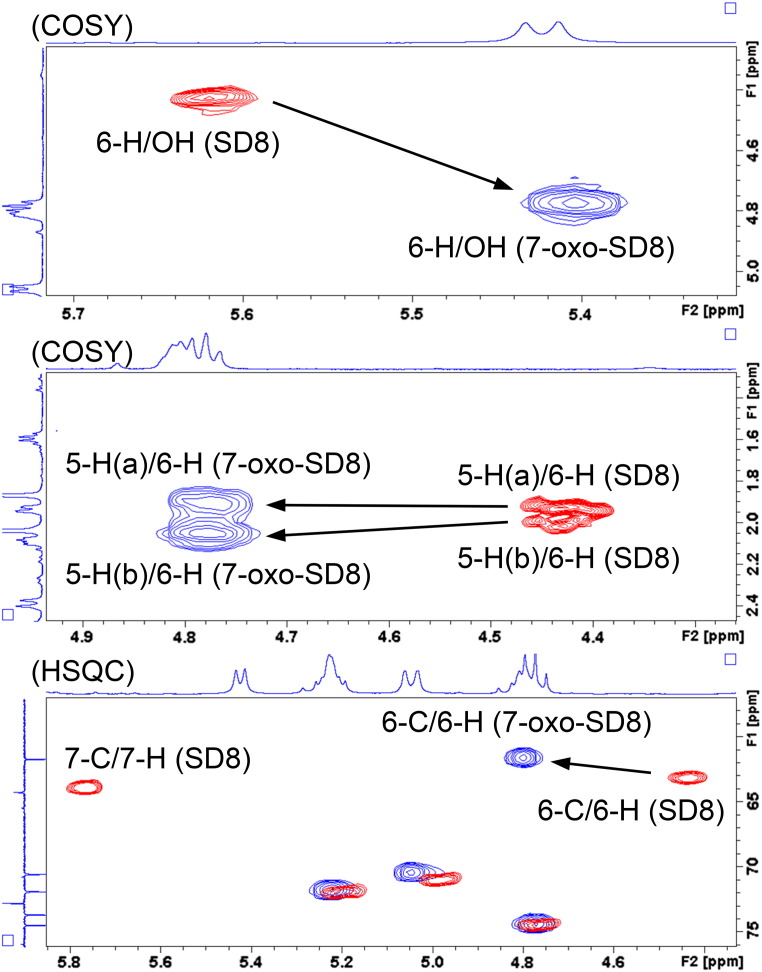
Shifted peaks and cross-peaks of SD8 (red) and 7-oxo-SD8 (blue) from 2D NMR (correlated spectroscopy and heteronuclear single quantum coherence). 2D NMR maps are shown with projections of proton and carbon signals from 7-oxo-SD8. These are entirely consistent with the introduction of a carbonyl group at the C-7 position.

Examination of the nuclear magnetic resonance (NMR) spectra [attached proton test, correlated spectroscopy, total correlated spectroscopy, heteronuclear single quantum coherence, and heteronuclear multiple bond correlation (HMBC)] suggested that the new molecule was structurally similar to SD8. However, the H-6 proton was shifted downfield by 0.4 ppm and no signal could be observed for the H-7 proton, consistent with a structural change in the angucyclic polyketide moiety (Fig. S4 and Table S1). Further analysis of the 2D (*2*-*d*imensional) NMR data enabled us to determine the position of the structural change (Figs. 4 and 5 and Figs. S5–S8). We confirmed appropriate proton–proton correlations for H-5 and H-6, which were supported by relevant proton–carbon correlations. In addition, we identified a signal shifted downfield for C-7 in the ^13^C spectrum typical for a carbonyl group (Figs. S9 and S10 and Table S2) and cross-peaks to a C-7 resonance could be identified in the HMBC experiments (Fig. 4 and Fig. S7). From this, we concluded that the product of the *simC7* mutant had a carbonyl group instead of a hydroxyl group at position C-7 in the angucyclic polyketide. The new simocyclinone intermediate was named 7-oxo-SD8 (Fig. 1).

### Complementation of the *simC7* mutant

To determine if the *simC7* phenotype was caused solely by loss of SimC7 function, we complemented the mutant with an *in trans* copy of the gene. *simC7* is the last gene of a putative seven-gene operon that starts with *simB7* (Fig. S1). Therefore, we drove expression of *simC7* from its putative native promoter by fusing *simC7* directly to an ~ 400-bp fragment spanning the intergenic region upstream of *simB7*, and we cloned this fusion into the vector pGM1190. Introduction of this construct restored SD8 production to the *simC7* mutant (data not shown).

### SimC7 is an NAD(P)H-dependent ketoreductase that acts on the angucyclic polyketide

**Fig. 6:**
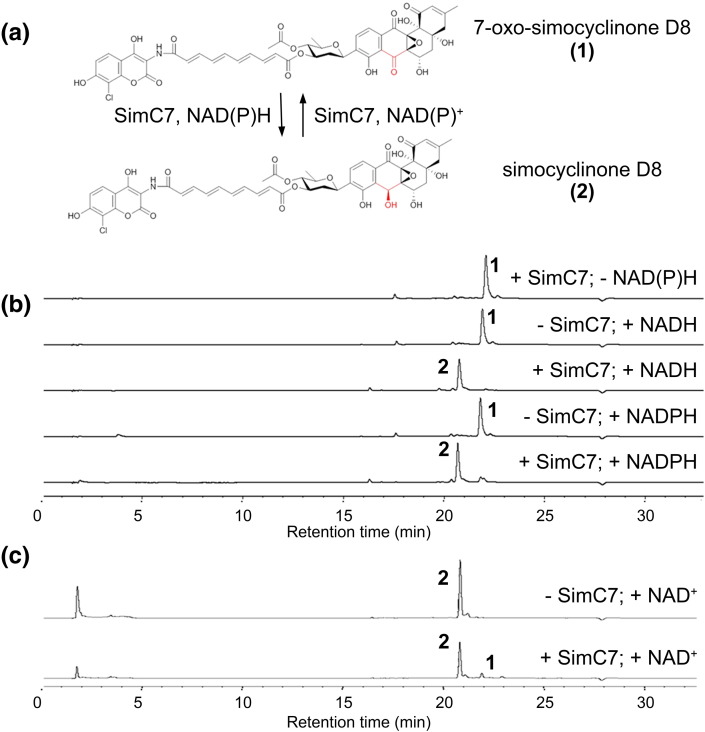
Ketoreductase activity of SimC7. (a) SimC7 mediates conversion of the novel simocyclinone intermediate 7-oxo-SD8 (1) into SD8 (2). (b) NAD(P)H-dependent conversion of 7-oxo-SD8 into SD8 and (c) NAD(P)^+^-dependent conversion of SD8 into 7-oxo-SD8, monitored by reversed-phase HPLC. Samples were incubated for 1 h at room temperature. Reactions were stopped by quenching in methanol (1:1) and heat denaturation for 10 min at 100 °C.

The fact that the *simC7* mutant made 7-oxo-SD8 suggested that SimC7 is a ketoreductase involved in biosynthesis of the angucyclic polyketide moiety and is not a dehydratase that acts during formation of the tetraene linker as had been proposed previously [11]. To test this hypothesis, we overexpressed an N-terminally His-tagged version of SimC7 in *E. coli* and assayed the purified recombinant protein for its ability to use 7-oxo-SD8 as a substrate. Both the substrate and the product of the reaction have strong chromophores with only minor differences in their absorbance spectra, and because their spectra also overlap those of NAD(P)^+^/NAD(P)H, it was not possible to assay SimC7 using spectrophotometric methods. Instead, we followed the reaction by HPLC-UV (with SD8 as a standard) and found that SimC7 readily converted 7-oxo-SD8 into SD8 (Fig. 6b). SimC7 was able to use NADH or NADPH for the reduction (Fig. 6b) but showed a preference for NADPH (data not shown). It was also possible to assay the reaction in the reverse direction, following the oxidation of SD8 to 7-oxo-SD8 by SimC7 (Fig. 6c). This reaction was much slower and required a very high concentration of the NAD^+^ cofactor (300 mM). In all cases, the identity of the reaction product was confirmed by tandem mass spectrometry.

### The C-7 hydroxyl group is required for the antibiotic activity of simocyclinone

The activity of 7-oxo-SD8 was tested *in vivo* and *in vitro*. Wild-type *E. coli* and other Gram-negative bacteria are resistant to simocyclinones because the compounds cannot penetrate the outer membrane [1]. We therefore employed an *E. coli* strain (NR698) that is sensitive to simocyclinones due to an in-frame deletion in the *imp* (*i*ncreased *m*embrane *p*ermeability) gene [22]. The MIC for SD8 was 0.3 μM. In contrast, NR698 grew in the presence of 7-oxo-SD8 at concentrations up to 17.5 μM, an ~ 60-fold increase in MIC.

**Fig. 7:**
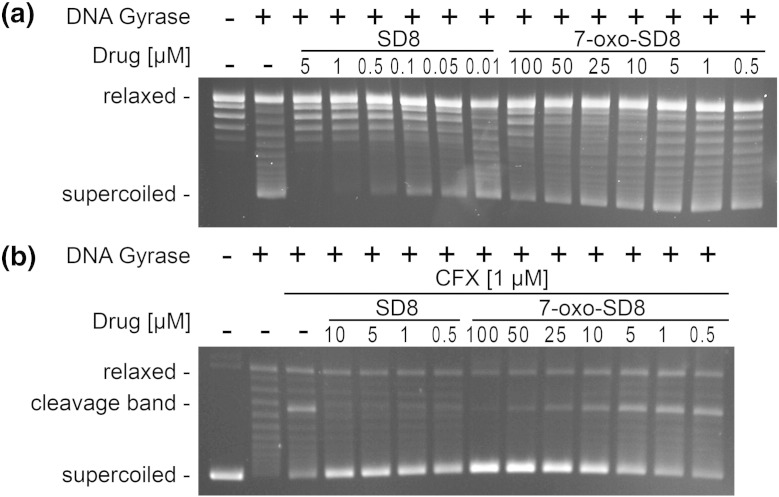
(a) Supercoiling and (b) cleavage relaxation assays with DNA gyrase in the presence of 7-oxo-SD8 or SD8. The reaction mixtures contained *E. coli* DNA gyrase and varying concentrations of either SD8 or 7-oxo-SD8. For relaxation assays, the mixtures were incubated without ATP and with ciprofloxacin (1 μM).

The contrasting antibiotic activities of SD8 and 7-oxo-SD8 against whole cells suggested that the oxidation state of the oxygen at the C-7 position might be important for its ability to block DNA supercoiling by DNA gyrase. We therefore tested the activity of SD8 and 7-oxo-SD8 as DNA gyrase inhibitors *in vitro*. In line with previous studies [7,23,24], we found that SD8 inhibited supercoiling by DNA gyrase with IC_50_ = 0.1–0.5 μM (Fig. 7a). In contrast, 7-oxo-SD8 was almost 3 orders of magnitude less active (IC_50_ = 50–100 μM) (Fig. 7a).

The fluoroquinolone ciprofloxacin links DNA gyrase to its substrate by stabilizing the DNA–protein cleavage complex, leading to a characteristic “cleavage band” on gels (Fig. 7b) [4]. The presence of SD8 blocks DNA cleavage by gyrase by preventing the enzyme from binding to DNA [7]. We tested the ability of 7-oxo-SD8 to abrogate the ciprofloxacin-stimulated cleavage of DNA by gyrase, using SD8 as a control (Fig. 7b) [6]. As we found previously [7], SD8 prevented DNA cleavage at low concentrations (e.g., 0.5 μM), but we found that much higher concentrations of 7-oxo-SD8 (> 10 μM) were required for inhibition of cleavage (Fig. 7b).

To investigate the binding of 7-oxo-SD8 to GyrA, we used surface plasmon resonance as described previously [7]. We found that SD8 bound to the GyrA N-terminal domain with a similar affinity to that reported previously, but that only weak, non-specific binding could be seen for the 7-oxo analog (data not shown). Taken together, these data suggest that 7-oxo-SD8 binds gyrase 2–3 orders of magnitude more weakly than SD8.

MGD8N2A is a simocyclinone analog (generated by chemical hydrolysis of SD8) that lacks the angucyclic polyketide (Fig. 1). This analog has been tested previously and found to have greatly reduced activity against DNA gyrase (IC_50_ = 50 μM) in comparison to SD8 (IC_50_ = 0.1–0.6 μM) [7]. We repeated these experiments with MGD8N2A and obtained a similar value (IC_50_ = 25 μM). Thus, comparing the IC_50_ values for 7-oxo-SD8 and MGD8N2A, it is clear that the presence of a carbonyl group at the C-7 position has a similarly negative effect on the activity of simocyclinone as does complete loss of the angucyclic polyketide.

## Discussion

To examine the prediction that SimC7 is a dehydratase involved in the biosynthesis of the tetraene linker, we isolated the ~ 72-kb *sim* gene cluster on a single PAC clone and expressed simocyclinone heterologously in an *S. coelicolor* strain engineered for improved antibiotic production. Deletion of *simC7* from the PAC clone resulted in the production of a novel simocyclinone, 7-oxo-SD8, which unexpectedly carried a normal tetraene linker but was altered in the angucyclinone moiety. We went on to demonstrate that SimC7 is an NAD(P)H-dependent ketoreductase that converts 7-oxo-SD8 into SD8 and that reduction of the keto group, catalyzed by SimC7, is essential for the antibiotic activity of simocyclinone.

### Use of the *S. coelicolor* M1152 heterologous expression system

Heterologous expression is a convenient tool for structure–function analysis of secondary metabolites, and improved derivatives of *S. coelicolor* and *Streptomyces avermitilis* have recently been developed for this purpose [21,25,26]. Here, we demonstrate the utility of this approach for the manipulation and functional analysis of simocyclinone, an antibiotic naturally made by *S. antibioticus* Tü6040, a strain that is particularly challenging for genetic manipulation. Despite the large size of the *sim* gene cluster (49 genes covering ~ 72 kb), it was straightforward to isolate a PAC clone carrying the entire cluster and to introduce it by conjugation into an engineered *S. coelicolor* host, where the clone integrated irreversibly and replicated stably as part of the chromosome. The same approach was recently used for the successful heterologous expression in *S. coelicolor* of the 83.5-kb FK506 gene cluster from *Streptomyces tsukubaensis* [20]. The engineered *S. coelicolor* host often gives a greater yield than the natural producer [20,21], and this was also true for simocyclinone. A further advantage of this approach is that genetic modification of the target cluster can be carried out in *E. coli*, giving full access to the advanced recombineering tools available in this host. The explosion of whole genome sequencing has revealed tens of thousands of new secondary metabolic gene clusters [27,28], many in rare and difficult-to-culture actinomycetes for which no genetic tools have been developed. As a consequence, the heterologous expression approach exemplified here is an attractive option for characterizing many of these clusters and is likely to be of increasing importance in the future.

### The biochemical function of SimC7

SimC7 was proposed to be a dehydratase involved in the biosynthesis of the tetraene linker [11], but we have demonstrated that SimC7 is actually an NAD(P)H-dependent ketoreductase that reduces a carbonyl group at the C-7 position of the angucyclic polyketide moiety. The fermentation product of the *simC7* mutant, 7-oxo-SD8, was almost inactive as a DNA gyrase inhibitor, showing that reduction of the 7-oxo functional group by the ketoreductase activity of SimC7 is essential for the biological function of simocyclinones. Although SimC7 readily converts 7-oxo-SD8 into SD8, synthesis of the angucyclic polyketide is normally completed before it is linked to the d-olivose sugar and the other moieties of simocyclinone [10]. This means that the natural substrate of SimC7 would be an A-group intermediate (i.e., one having only the angucyclic polyketide moiety) carrying a C-7 carbonyl. This species was detected neither in the native producer *S. antibioticus* [10–12] nor in the heterologous host carrying the wild-type *sim* cluster, but as expected, a species with the appropriate mass was detected among the intermediates made by the *simC7* mutant. Having determined the true function of SimC7, it remains unclear how the tetraene linker of simocyclinone is assembled and how dehydration takes place.

SimC7 (30 kDa; 284 amino acids) is a member of the SDR family, one of the largest protein superfamilies, having more than 120,000 representatives in the databases. SDR proteins are diverse, with low overall amino acid sequence identity (typically 20–30% in pairwise comparisons), and they are principally characterized by the presence of a predicted pyridine nucleotide-binding Rossmann fold, comprising a parallel β-sheet flanked by three helices on each side. SDR proteins also have diverse biochemical activities, including acting as dehydratases, reductases, dehydrogenases, decarboxylases, and epimerases [29,30]. Despite its function as an angucyclinone ketoreductase, based on sequence identity, SimC7 seems to be most similar to various SDR sugar epimerases, and SimC7 secondary and tertiary structure predictions using the Phyre2 server [31] also show highest similarity to sugar-modifying enzymes, such as epimerases, reductases, and dehydratases. No close homologs in the databases are known to function as ketoreductases.

Although none is closely related to SimC7, several SDR ketoreductases have been identified that act on polyketides. These include SDR proteins that reduce the carbonyl group at position C-9 in the biosynthetic pathways for jadomycin (JadE), actinorhodin (ActKR), and hedamycin (HedKR) [32–34] and the SDR protein that reduces the carbonyl group at position C-6 in the landomycin pathway (LanV) [35]. Close homologs of these enzymes are encoded in the *sim* gene cluster, raising the possibility that SimA6 and SimA9 function to reduce the angucyclinone C-10 and C-6 carbonyl groups, respectively, during the biosynthesis of simocyclinone.

### SimC7 function is vital for producing simocyclinones with antibiotic activity

**Fig. 8:**
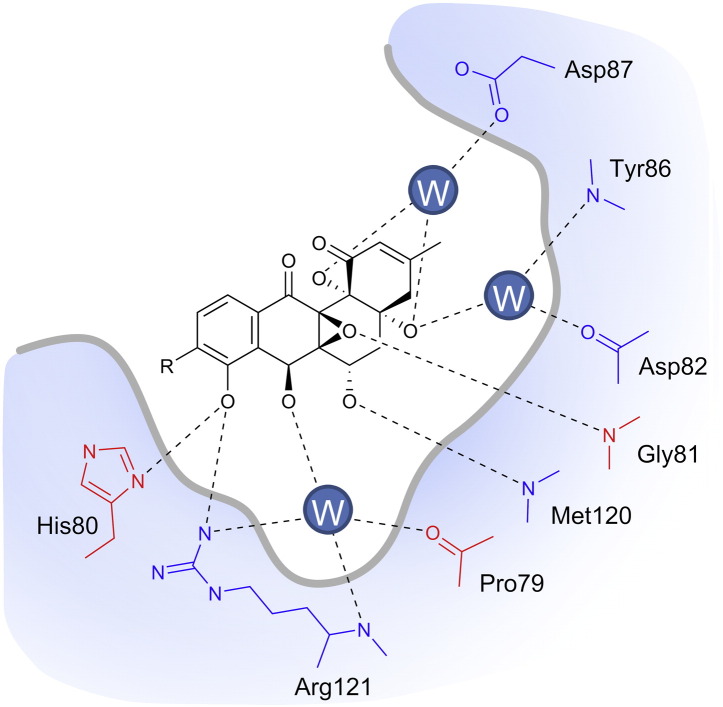
The angucyclic polyketide binding pocket in GyrA, as revealed in the crystal structure of the GyrA–SD8 complex (PDB accession number 4CKL) [8]. SD8 is contacted by residues from both GyrA subunits; those from one subunit are shown in red and those from the other subunit are shown in blue. For clarity, all hydrogens have been omitted.

Why does such a small structural change render 7-oxo-SD8 effectively inactive as a DNA gyrase inhibitor? The crystal structure of the GyrA–SD8 complex revealed how DNA gyrase binds the angucyclic polyketide [8] (Fig. 8). The C-6 hydroxyl group makes a hydrogen bond to Met120, the C-7 hydroxyl group makes hydrogen bonds to Pro79 and Arg121 (via a water molecule), and the C-8 hydroxyl group makes hydrogen bonds to His80 and Arg121. One speculative possibility is that the presence of a C-7 carbonyl group in 7-oxo-SD8 leads to the formation of intramolecular hydrogen bond with the neighboring C-8 hydroxyl group, thus simultaneously breaking contacts with His80 and a highly coordinated water molecule held in place by Pro79 and Arg121 (Fig. 8). His80 in particular seems to play a crucial role in binding simocyclinone, as the SD8 IC_50_ is increased 230-fold when this residue is mutated to alanine [7]. In addition, the presence of a carbonyl group at C-7 will alter the conformation of the angucyclic polyketide, which may affect other bonding interactions with GyrA. Thus, the introduction of a carbonyl group at C-7 is likely to break several hydrogen bonds that secure the angucyclic polyketide in its binding pocket.

## Materials and Methods

### Strains, plasmids, and oligonucleotides

Strains, plasmids, and oligonucleotides used in this work are shown in Tables S3, S4, and S5, respectively.

### Antibiotic selection

Apramycin (50 μg/mL), carbenicillin (100 μg/mL), chloramphenicol (25 μg/mL), hygromycin (40 μg/mL), kanamycin (50 μg/mL), nalidixic acid (25 μg/mL), and thiostrepton (60 μg/mL) were added to solid media as required unless otherwise noted. These concentrations were halved in liquid cultures.

### PAC library construction

A genomic library of *S. antibioticus* Tü6040 DNA was constructed by Bio S&T Inc. (Montreal, Canada) as described in Ref. [20], using the phage P1-derived artificial chromosome (PAC) vector pESAC13†, a derivative of pPAC-S1 [36]. The integrative vector pESAC13 conferred kanamycin resistance in *E. coli* and thiostrepton resistance in *Streptomyces*. Genomic DNA was isolated and cloned between the two BamHI sites of pESAC13, replacing the carbenicillin resistance gene. The genomic DNA library consisted of 2688 individual PAC clones with an average insert size of 110 kb (more than 20 × genome coverage).

### Identification of PAC clones containing the complete *sim* gene cluster

The *S. antibioticus* PAC library was screened with two primer pairs (Table S5) amplifying fragments flanking the *sim* gene cluster. The screening identified six double-positive clones with amplification of both PCR products. One double-positive clone (PAC-12I; Table S4) was sequenced. The insert in PAC-12I contained the minimal 72-kb *sim* gene cluster flanked by genomic regions of ~ 19 kb and 4 kb on either side (a total insert size of 95 kb), and PAC-12I was chosen for heterologous expression studies.

### Deletion of *simC7* from PAC-12I and *in trans* complementation

The *simC7* gene was deleted by PCR targeting according to standard protocols [37,38], leaving an 81-bp in-frame scar. The PAC mutant (PAC-12IΔC7) structure was confirmed by PCR and restriction digestion with EcoRI. For complementation, *simC7* was fused to the *simB7* promoter and cloned into pGM1190. Complementation of *simC7* was confirmed by liquid chromatography mass spectrometry (LC-MS).

### Mobilization of PAC clones

PAC clones were conjugated from *E. coli* DH5α into the methylation-deficient *E. coli* strain ET12567 by triparental mating, using *E. coli* TOP10 cells carrying the driver plasmid pR9406 to mobilize the PAC [20]. PAC clones were conjugated from *E. coli* ET12567(pR9406) into *S. coelicolor* M1152 and M1152ex1 as previously described [39]. After 16–20 h of incubation on R2 medium without sucrose, plates were overlaid with thiostrepton and nalidixic acid. Exconjugants were streaked on Mannitol Soy Flour medium with thiostrepton and nalidixic acid. For spore preparation, strains were grown on Instant Mashed Potato Agar (2 g/L Smash® instant mashed potato and 2 g/L LabM agar, made up with tap water).

### Production medium and purification of simocyclinones

To optimize production of d-group simocyclinones, we cultured strains for 6 days at 30 °C and 250 rpm in the basal chemically defined medium described in Ref. [10], supplemented with l-glutamine (5.84 g/L) as nitrogen source and glycerol (20 mL/L) as carbon source. The fermentation broth was acidified with HCl (5 M) to pH 4 and simocyclinones were extracted with an equal volume of ethyl acetate. After centrifugation, the organic layer was evaporated *in vacuo*. For the purification of 7-oxo-SD8, the dried extract from 4 L culture medium (~ 1.5 g with ~ 15% compound) was dissolved in methanol, filtered through glass wool and purified by normal-phase liquid chromatography on a silica column (Biotage^®^ SNAP cartridge KP-SIL 50 g, 40- to 65-μm particle size, 39 mm × 81 mm) using a gradient elution from CH_2_Cl_2_-MeOH (95:5) to CH_2_Cl_2_-MeOH (70:30) over 4 h at a flow rate of 50 mL/h [40]. Fractions of 10 mL were collected and the presence of the compound was verified by thin layer chromatography and LC-MS. Selected fractions were pooled, dried *in vacuo*, and tritiated in acetone. The acetone-soluble fraction was dried and dissolved in methanol for size-exclusion chromatography using Sephadex LH-20 (18- to 111-μm particle size) (Sigma-Aldrich). Following elution using methanol, we collected fractions and confirmed the presence of the compound by LC-MS. Fractions containing 7-oxo-SD8 were combined, the solvent was removed under reduced pressure, and the concentrate was dissolved in a methanol–acetone mix (1:1) and purified further by preparative HPLC. After freeze drying, we obtained ~ 63 mg of the new simocyclinone derivative in a fine yellow powder (yield, ~ 27%; purity, > 98%).

### High-performance liquid chromatography

For analytical HPLC, samples (30 μL) were separated on an HPLC column (Phenomenex Gemini-NX 3u C_18_ 110A, 150 mm × 4.6 mm) using a linear gradient with 0.1% formic acid as mobile phase A and methanol as mobile phase B. The gradient was either 25–95% or 75–95% solvent B over 20 min at a flow rate of 1 mL/min. Absorbance was recorded with a diode array detector at 210 nm, 230 nm, and 360 nm for simocyclinones and 535 nm as reference. For preparative HPLC, samples (350 μL) were injected onto an HPLC column (Phenomenex Gemini-NX 5u C_18_ 110A AXIA Packed, 150 mm × 21.20 mm) with a Phenomenex SecurityGuard Prep Cartridge Holder Kit (21.20 mm) attached to a Dionex UltiMate 3000 HPLC machine. Separation was performed using a linear gradient with 0.1% formic acid as mobile phase A and methanol as mobile phase B. The gradient was 75–95% mobile phase B over 20 min at a flow rate of 21 mL/min, and absorbance was recorded at 360 nm.

### Liquid chromatography mass spectrometry

Samples (10 μL) were injected onto an HPLC column (Phenomenex Luna-C_18_ 3u C18(2) 100A, 100 mm × 2 mm) with a Phenomenex SecurityGuard Prep Cartridge Holder Kit (2 mm), attached to a Thermo ion-trap LC-MS (LCQ DECA XPlus). Separation was performed using a linear gradient with 0.1% formic acid as mobile phase A and methanol as mobile phase B. The gradient was 25–95% mobile phase B over 20 min at a flow rate of 1 mL/min.

### NMR spectrometry

Spectra were recorded on a Bruker Avance DRX-700 and Avance III 400 spectrometer at 298 K. Data were processed and analyzed using TopSpin v3.2 software (Bruker). Assignments were made from experiments recorded on the DRX-700 spectrometer; ^1^H coupling constants were determined using experiments from the Avance III 400 spectrometer. Simocyclinones were dissolved in DMSO-*d*_6_.

### Overexpression and purification of SimC7

*simC7* was PCR amplified from PAC-12I using Q5 DNA polymerase (New England Biolabs) and oligonucleotides that introduced NdeI and BamHI sites (Table S5). The PCR product was cloned into pJET1.2/blunt and then sub-cloned into pET15b vector (Novagen) cut with NdeI and BamHI. The resulting construct, pET15b-NB-C7, was sequenced for errors (MWG Eurofins).

*E. coli* Rosetta (DE3)pLysS-competent cells (Novagen) were transformed with pET15b-NB-C7 and grown at 25 °C in LB containing chloramphenicol and carbenicillin. One 50-mL overnight culture was used to inoculate two 400-mL main cultures that were grown to an OD_600_ of ~ 0.4. Protein expression was induced with IPTG added to a final concentration of 0.5 mM and cultures were grown for an additional 16 h at 25 °C. Cells were harvested and lysed by sonication in 40 mL fresh lysis buffer [50 mM Tris–HCl (pH 8.0), 150 mM NaCl, 0.1% (v/v) Triton-X100, 0.5 mg lysozyme, and complete ethylenediaminetetraacetic-acid-free protease inhibitor (Roche)]. Cell debris was removed by centrifugation, the supernatant was incubated with 0.6 mL of Ni-NTA agarose beads (Qiagen) for 30 min, and then the resin was packed into a 5-mL polypropylene column (Qiagen). Unbound proteins were removed with 200 mL wash buffer [50 mM Tris–HCl (pH 8.0), 250 mM NaCl, and 5% (v/v) glycerol] and His-tagged SimC7 was eluted in 2–3 mL elution buffer [50 mM Tris–HCl (pH 8.0), 50 mM NaCl, 5% (v/v) glycerol, and 250 mM imidazole]. The purified protein was dialysed overnight into storage buffer [20 mM Tris–HCl (pH 8.0), 150 mM NaCl, 5% (v/v) glycerol, 1 mM DTT, and 2 mM ethylenediaminetetraacetic acid] and then frozen in liquid nitrogen and stored at − 80 °C. Protein concentrations were determined using Bradford reagent (BioRad).

### SimC7 ketoreductase assays

Ketoreductase assays were performed in reaction mixtures consisting of substrate (25–200 μM), NAD(P)H (0.3 mM) or NAD^+^ (300 mM), Hepes (50 mM, pH 7.2), and enzyme (500 nM). Reactions were initiated with SimC7 and quenched at specific time points with equal volumes of methanol. Stopped reactions were incubated for 10 min at 100 °C and centrifuged to remove precipitated protein (13,000*g* for 10 min at 4 °C). SD8 formation was quantified by analytical reversed-phase HPLC using a linear gradient of 25–95% methanol over 20 min then 95% methanol for 5 min against 0.1% formic acid in water, and absorbance was recorded at 360 nm. SD8 concentrations were calculated by comparison with a standard curve (Fig. S11), which was calculated from a serial dilution of SD8 prepared in methanol (0.25–70 μM) under the conditions described above (7.5–2100 pmol).

### Minimum inhibitory concentrations

MICs were determined as described in Ref. [41]. *E. coli* NR698 [22] was grown in Müller-Hinton broth for 16–20 h at 37 °C and bacterial growth was measured photometrically at 600 nm wavelength.

### DNA gyrase assays

Assays were performed as described previously [6,8].

## Footnotes

† http://www.ncbi.nlm.nih.gov/nuccore/677282244
