# Supplementary material for: SimC7 Is a Novel NAD(P)H-Dependent Ketoreductase Essential for the Antibiotic Activity of the DNA Gyrase Inhibitor Simocyclinone
Source: J Mol Biol. 2015 Jun 19;427(12):2192–204. doi: 10.1016/j.jmb.2015.03.019 (PMC4451461; doi:10.1016/j.jmb.2015.03.019)
Supplement: Supplementary file 1 — Supplementary material. [file mmc1.pdf]

Supplementary data for:

SimC7 is a novel NAD(P)H-dependent ketoreductase essential for the antibiotic activity of the DNA gyrase inhibitor simocyclinone

Martin Schäfer, Tung B.K. Le, Stephen J. Hearnshaw, Anthony Maxwell, Gregory L. Challis, Barrie Wilkinson and Mark J. Buttner

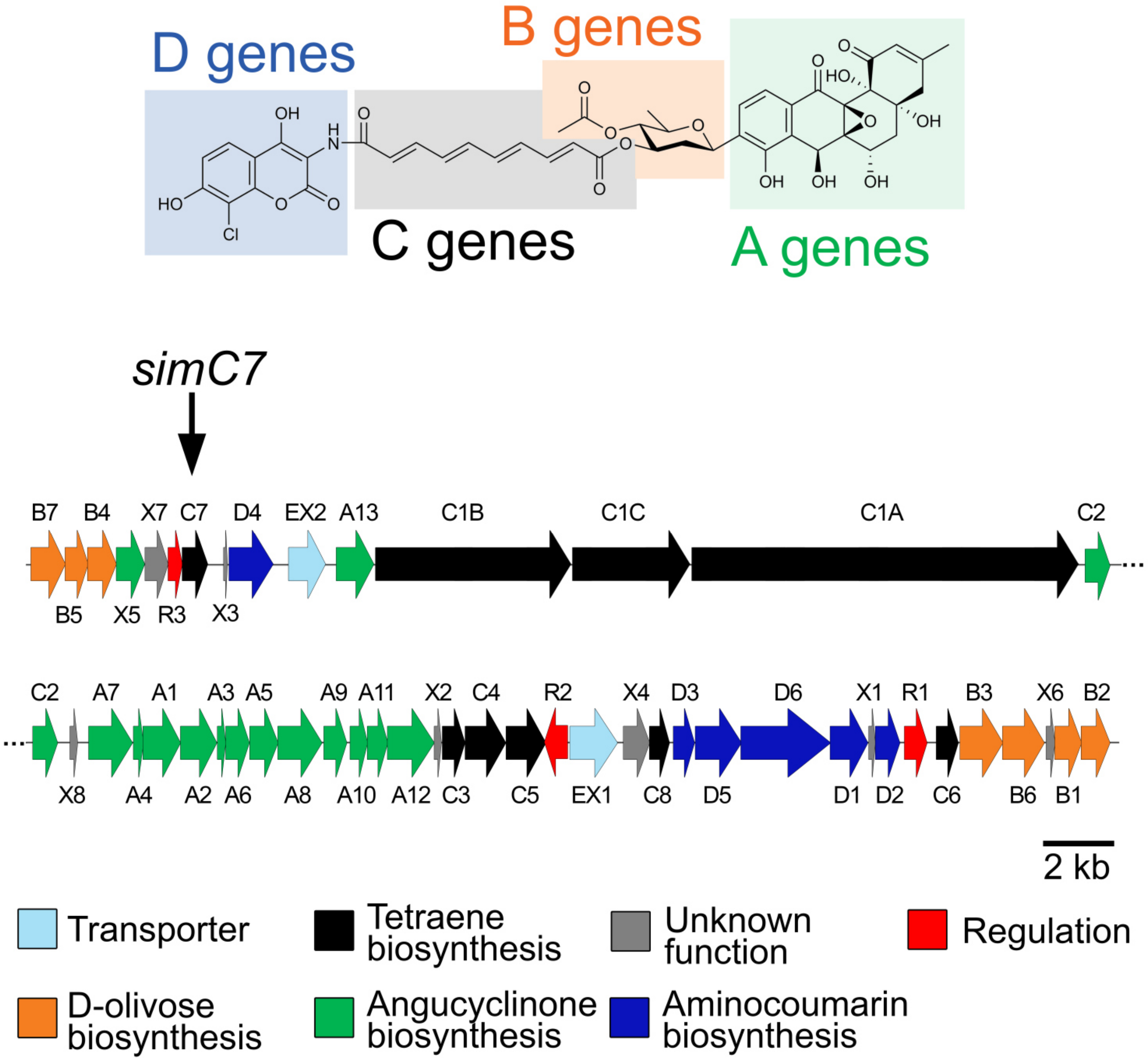

Fig. S1. The simocyclinone (*sim*) biosynthetic gene cluster. Genes are colour-coded according to their proposed involvement in the biosynthesis of the four moieties of simocyclinone [1].

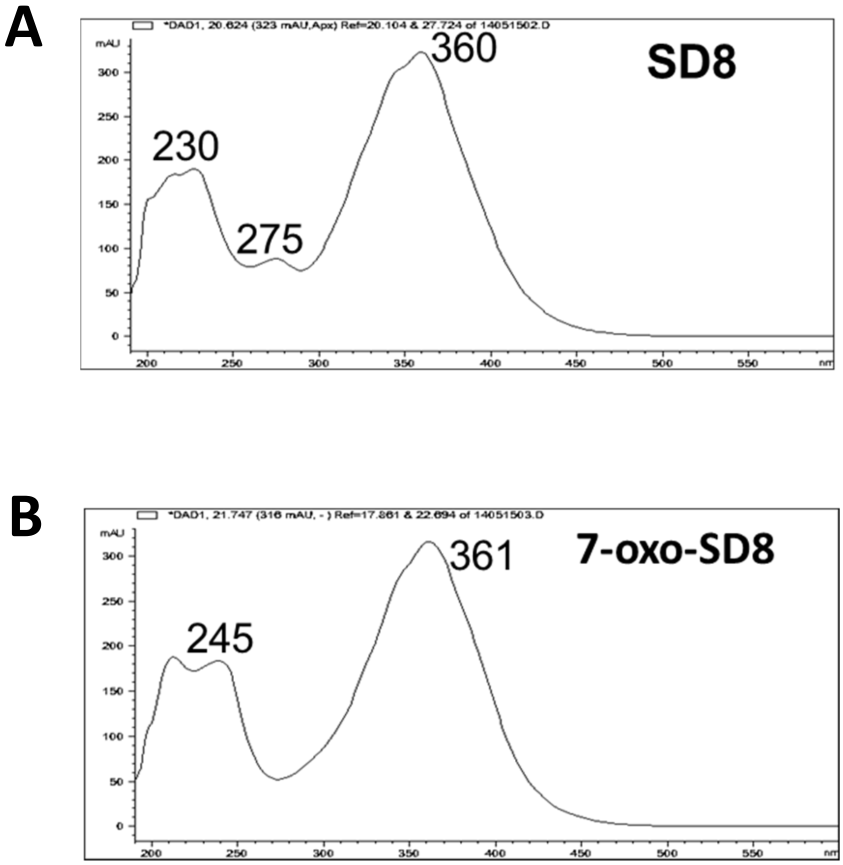

Fig. S2. Absorbance spectra of (A) SD8 and (B) 7-oxo-SD8 with indicated absorbance maxima.

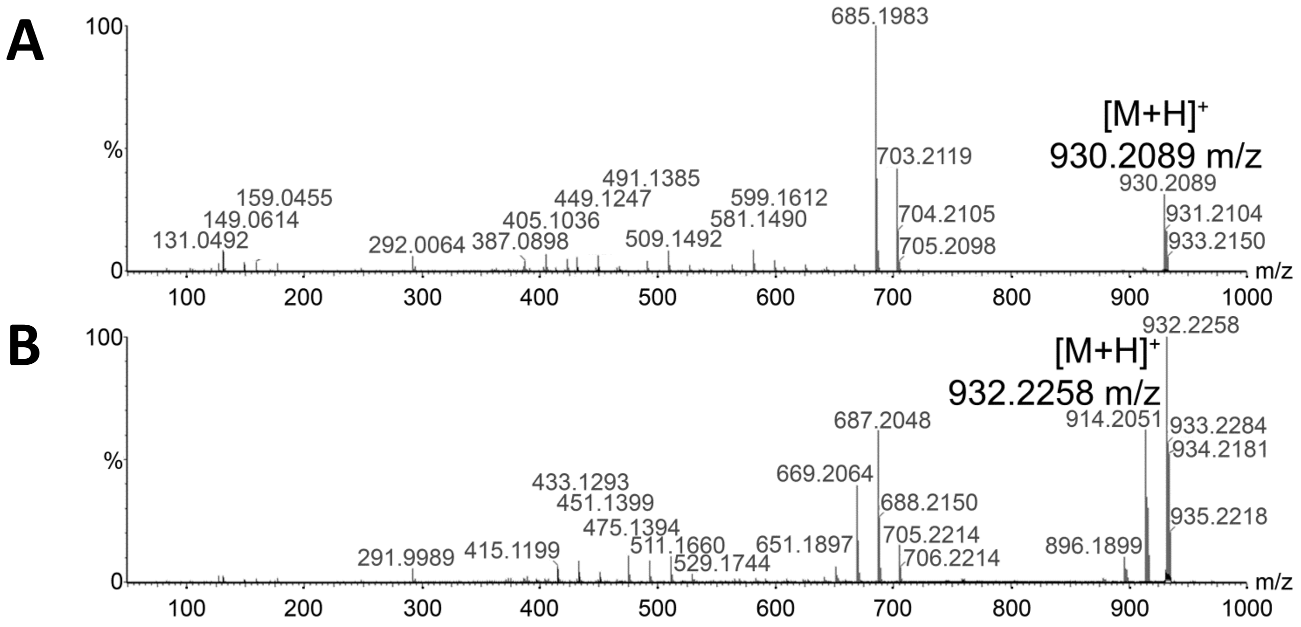

Fig. S3. High resolution mass spectrometry of (A) 7-oxo-SD8 and (B) SD8.

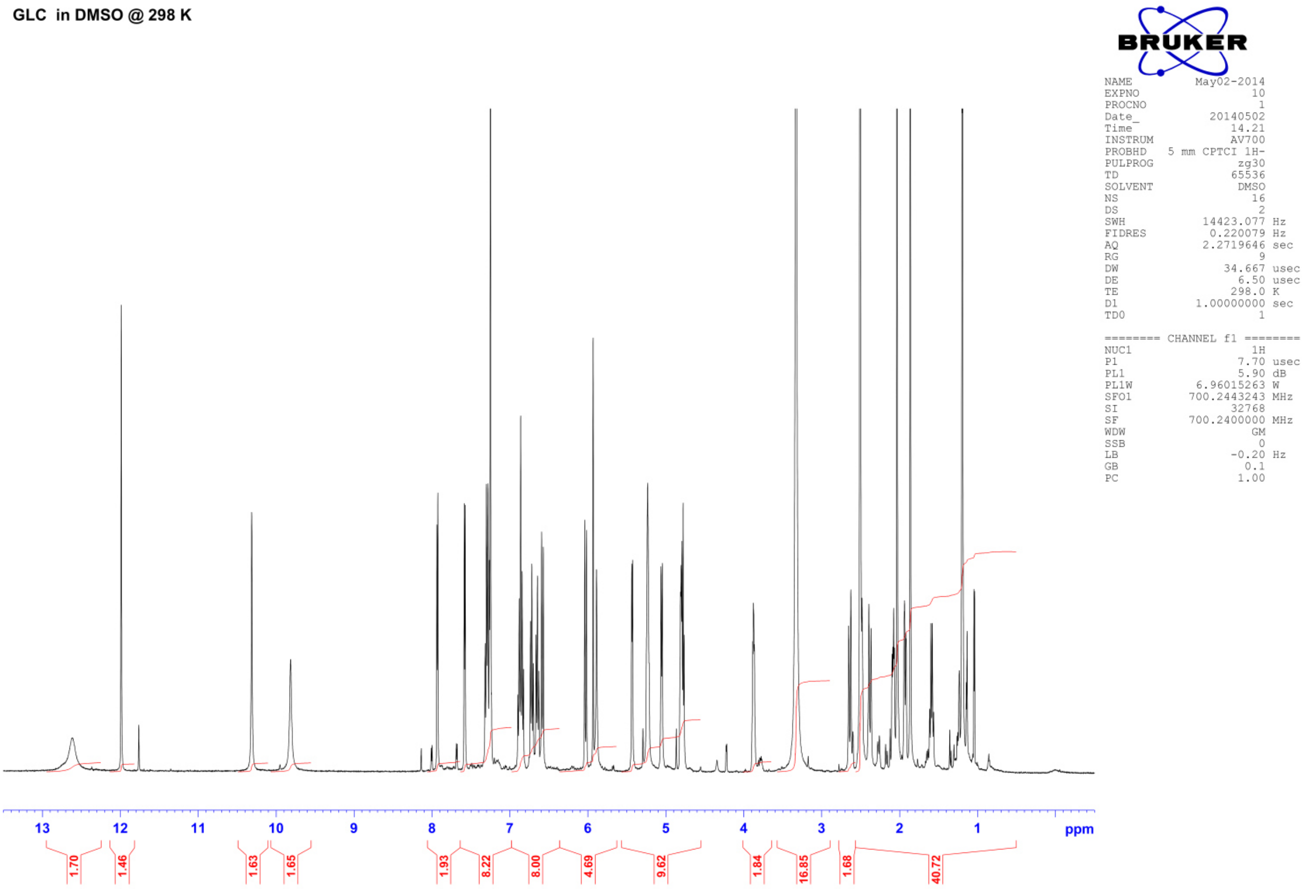

Supp. Fig. 4. ^{1}H NMR spectrum of 7-oxo-SD8.

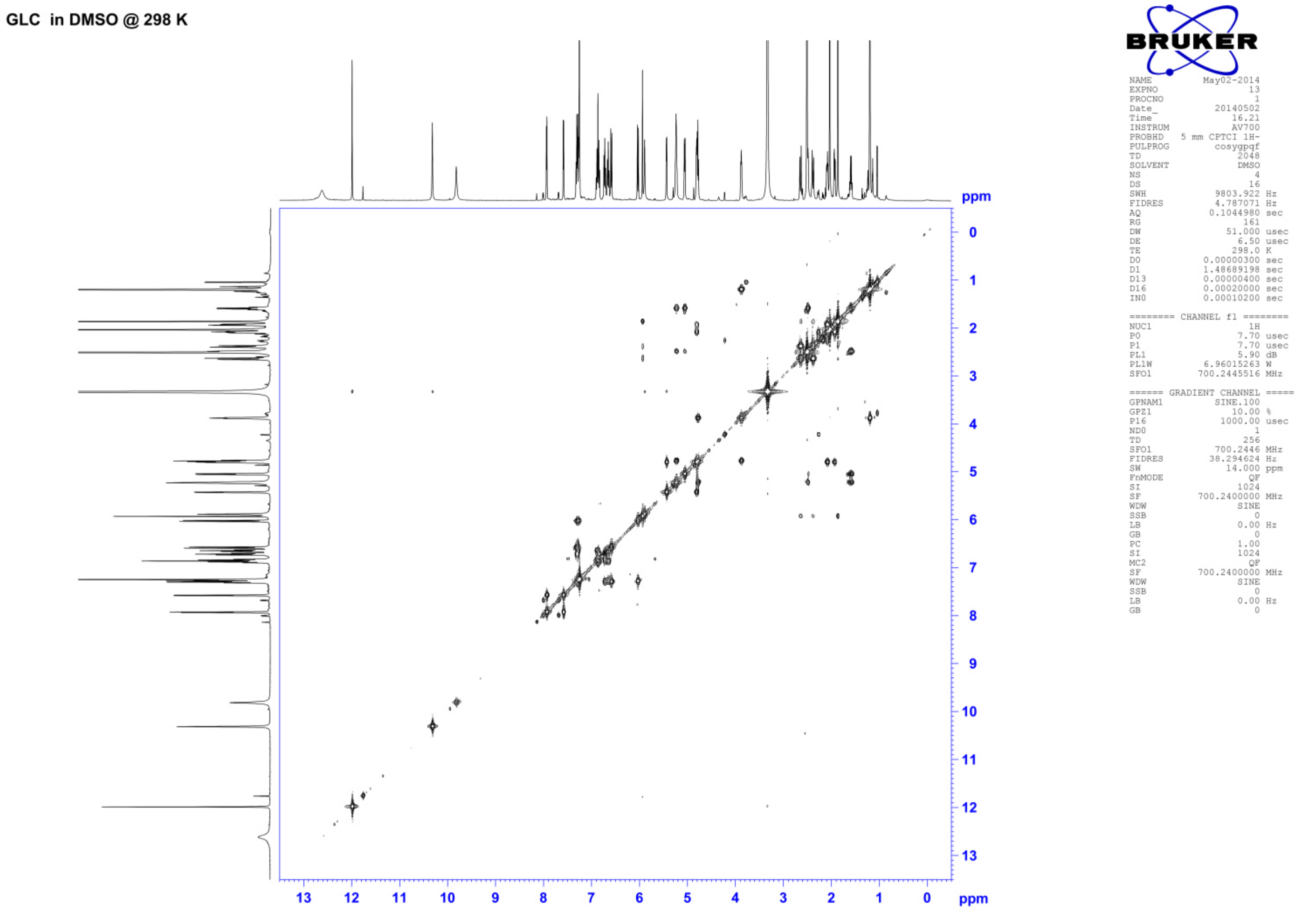

Fig. S5. Correlated spectroscopy (COSY) spectrum of 7-oxo-SD8.

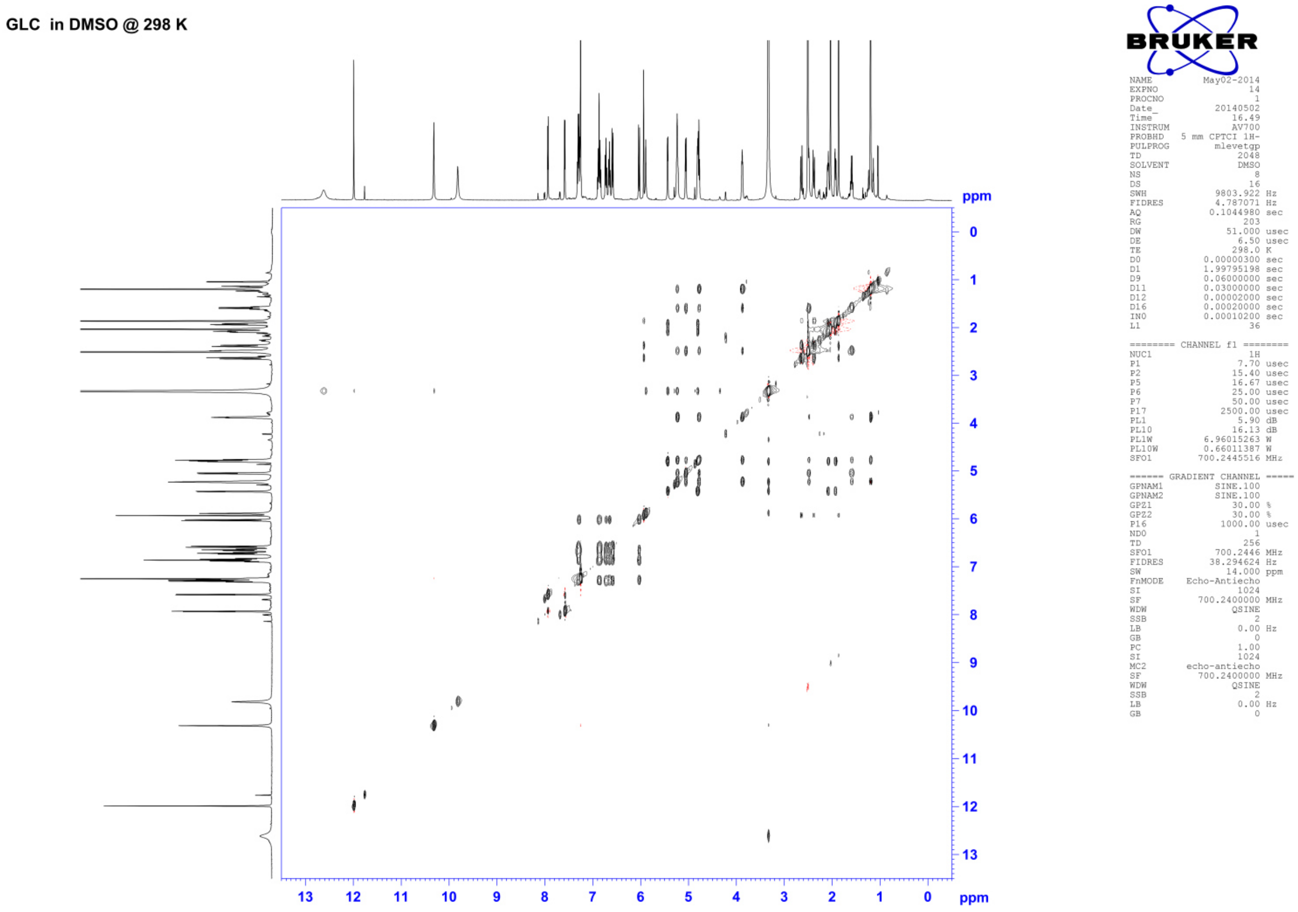

Fig. S6. Total correlated spectroscopy (TOCSY) spectrum of 7-oxo-SD8.

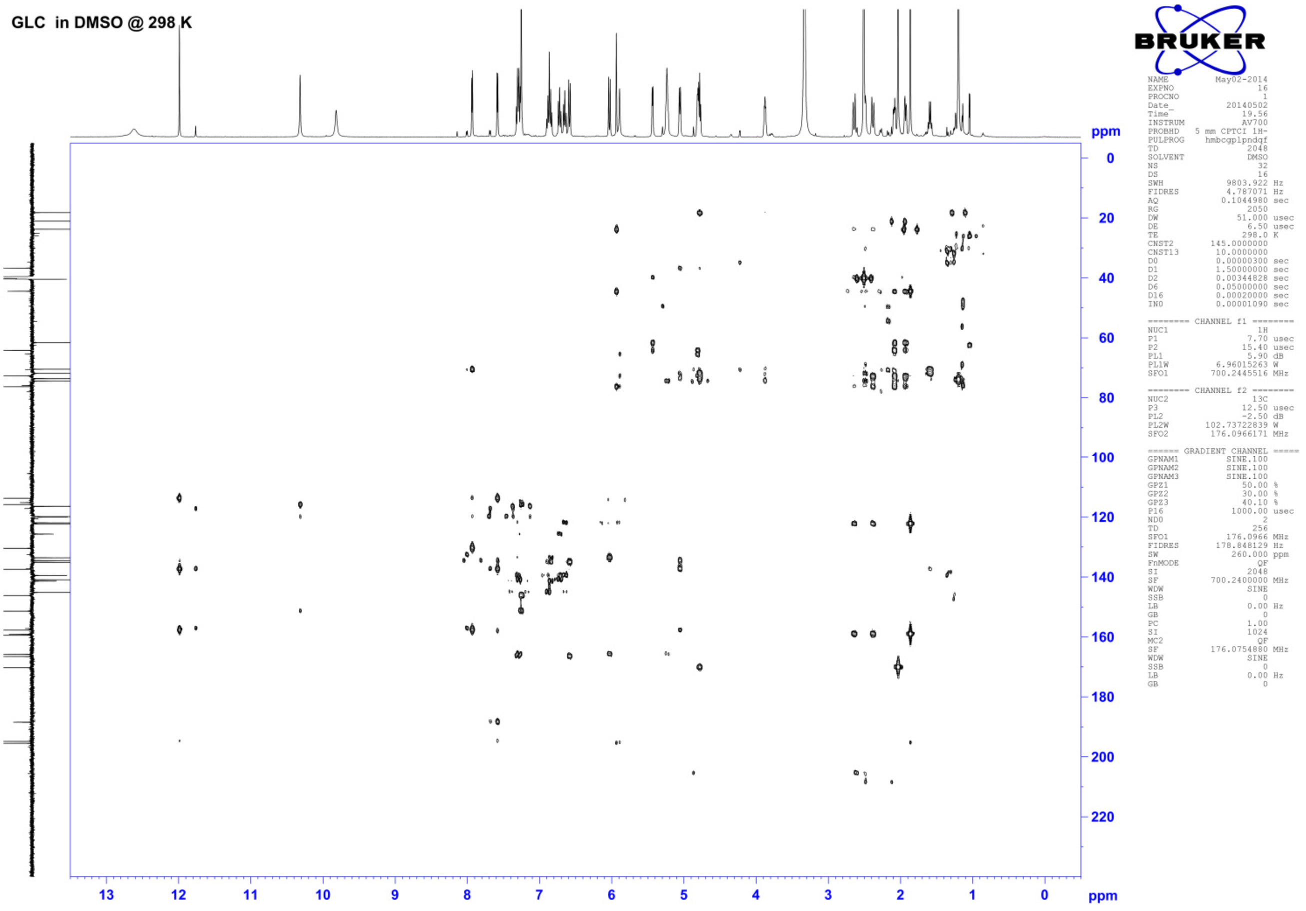

Fig. S7. Heteronuclear multiple bond correlation (HMBC) spectrum of 7-oxo-SD8.

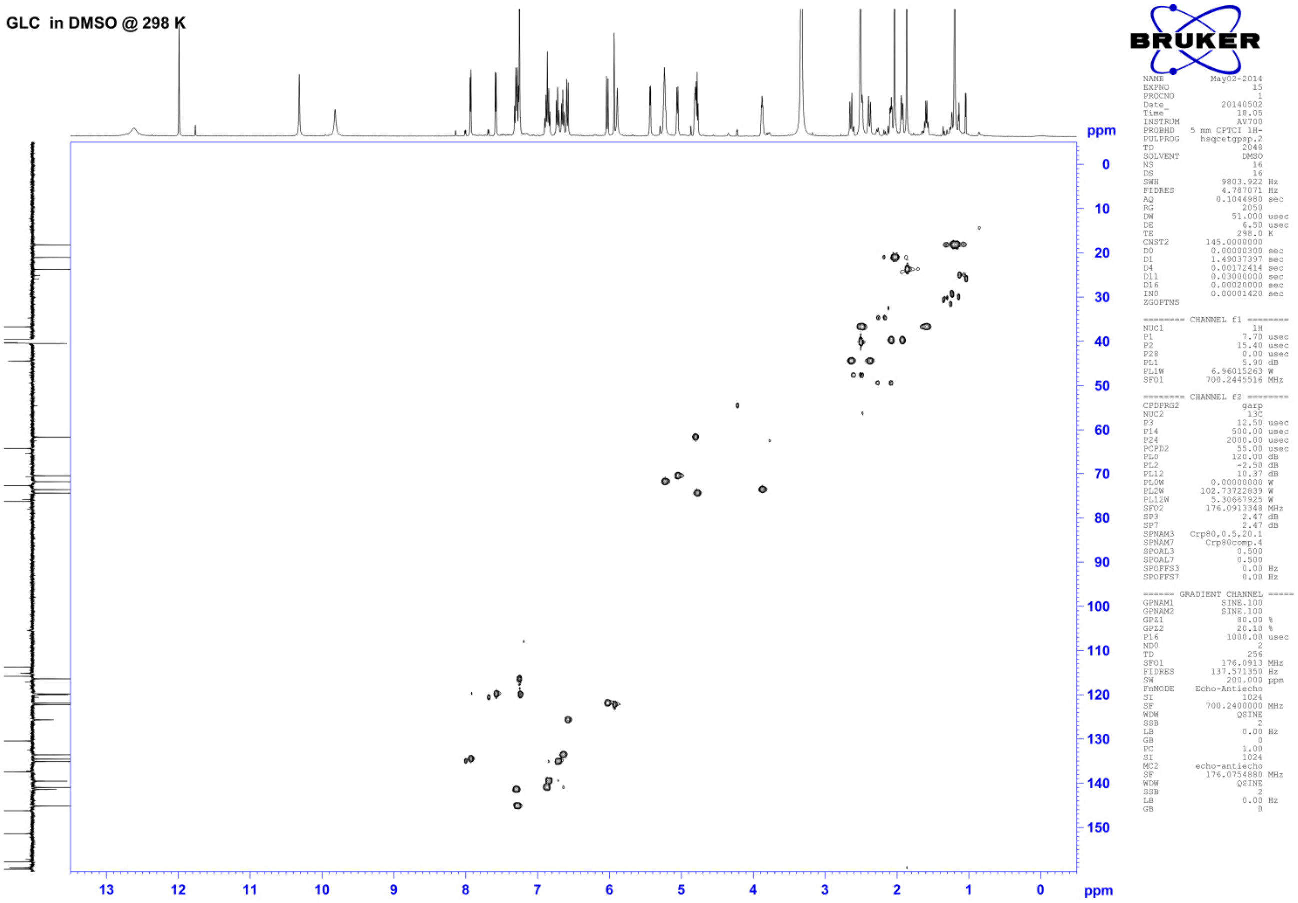

Fig. S8. Heteronuclear single quantum coherence (HSQC) spectrum of 7-oxo-SD8.

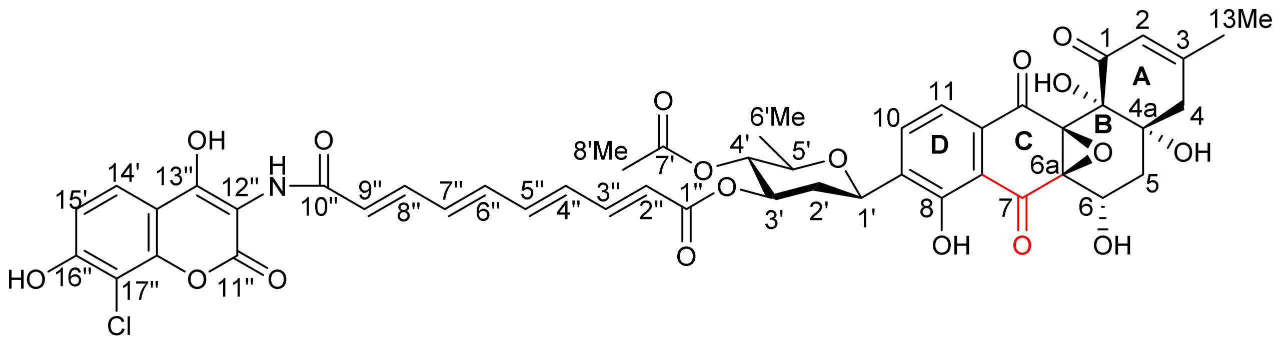

Figure S9. Numbering of carbon atoms in 7-oxo-SD8 for nuclear magnetic resonance (see Tables S1 and S2).

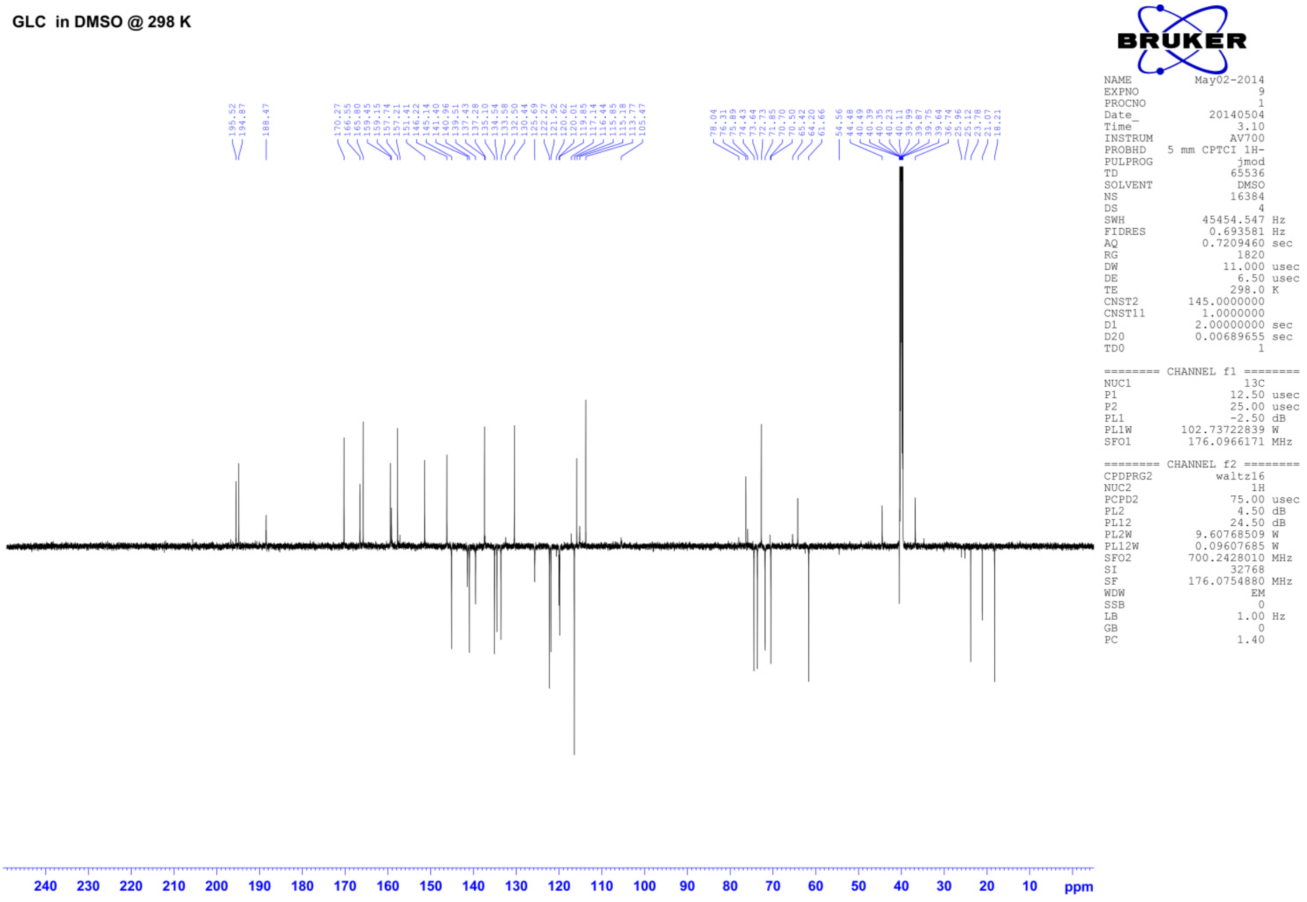

Supp. Fig. S10. Attached proton test (APT) ^{13}C-NMR spectrum of 7-oxo-SD8.

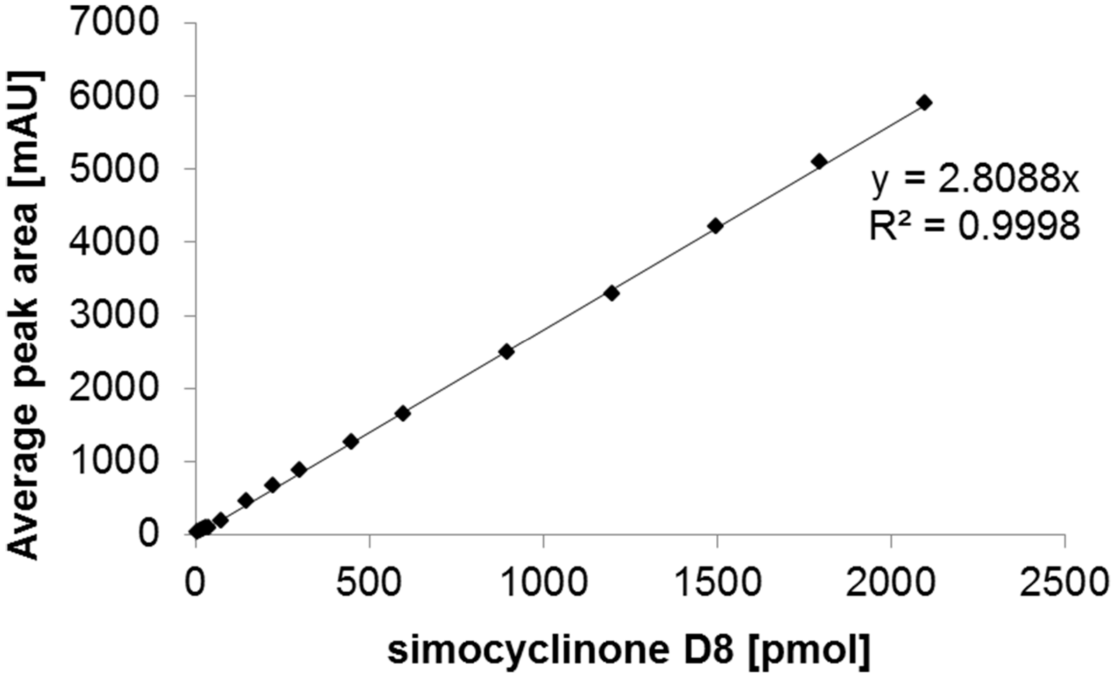

Fig. S11. SD8 standard curve for determination of product conversion by SimC7. Standards (7.5 - 2100 pmol) were analysed by HPLC in triplicate and the average peak area was determined. The coefficient of determination (R^2) was 0.9998.

Table S1

^{1}H NMR data (700 MHz) for 7-oxo-SD8 in d_6-DMSO. Data for SD8 were taken from [1] for comparison.

^{1}H NMR	SD8		7-oxo-SD8	
Proton(s)	δH (ppm)	δH (J in Hz)	δH (ppm)	δH (J in Hz)
6'-CH_3	1.16	d, 6.0	1.19	d, 6.02
2'-CH_2 (a)	1.55	ddd, 11.5, 11.5, 11.5 (ax)	1.59	dd, 11.73. 11.11
13-CH_3	1.81	s	1.86	s
5-H (a)	1.91	ddd, 13.5, 7.5, 1.0	1.92	dd, 14.59, 5.09
5-H (b)	1.98	m	2.08	m
8'-CH_3	2.01	s	2.03	s
4-CH_2 (a)	2.23	d, 19.0	2.38	d, 19.32
2'-CH_2 (b)	2.42	ddd, 1.0, 4.5, 11.5 (eq)	2.47	d, 5.65 (1)
4-CH_2 (b)	2.70	d, 19.0	2.64	d, 19.32
5'-H	3.78	dq, 6.0, 9.0 (ax)	3.87	dq, 6.09, 15.50
6-H	4.42	dd, 7.5, 7.5	4.82	m (2)
4'-H	4.74	dd, 9.0, 9.0 (ax)	4.77	dd, 9.41, 9.41
OH (a)	4.82		---	---
1'-H	4.96	dd, <1, 11.5 (ax)	5.05	dd, 0.94, 11.48
3'-H	5.18	ddd, 4.5, 9.0, 11.5 (ax)	5.23	m
12b-OH	5.27		5.88	s
6-OH	5.58		5.43	d, 7.78
7-H	5.75	s	---	---
2-H	5.90	s	5.93	q, 1.16 ($_2$J)
2''-H	6.00	d, 15.0	6.03	d, 15.06
9''-H	6.59	d, 15.0	6.58	d, 15.18
4''-H	6.61-6.73	m	6.60-6.77	m
7''-H	6.61-6.73	m	6.60-6.77	m
5''-H	6.79-6.88	m	6.80-6.92	m
6''-H	6.79-6.88	m	6.80-6.92	m
8''-H	7.23	m/ dd, 15.0, 11.0	7.23	m
14''-H	7.23	m	7.23	m
15''-H	7.23	m	7.23	m
3''-H	7.26	dd, 15.0, 11.0	7.23	m
11-H	7.33	d, 8.0	7.58	d, 7.91
10-H	7.45	d, 8.0	7.93	d, 8.03
OH (b)	9.58		9.81	s
OH (c)	9.85		10.31	s
NH	11.30		11.98	s
13''-OH	12.60		12.61	s

(1) Due to solvent overlap and poor resolution only a doublet observed

(2) Due to overlap with 4'-H signal could not extract J value.

Table S2

^{13}C NMR data (700 MHz) for 7-oxo-SD8 in d_6-DMSO. Data for SD8 were taken from [1] for comparison.

^{13}C NMR position	carbon	SD8 δH (ppm)	7-oxo-SD8 δC (ppm)
6'	CH_3	17.70	18.21
8'	CH_3	20.50	21.07
13	CH_3	23.10	23.78
2'	CH_2	36.50	36.74
5	CH_2	38.70	40.49
4	CH_2	42.70	44.48
6	CH	62.60	61.66
7	CH	63.50	194.87
6a	C	65.40	64.20
12a	C	65.70 (1)	65.40
1'	CH	70.60	70.50
3'	CH	71.60	71.85
4a	C	72.00	72.73
5'	CH	73.10	73.64
4'	CH	74.10	74.43
12b	C	75.10	76.31
12''	C	105.30	105.47
17''	C	114.40	113.77
13a''	C	115.30	115.85
15''	CH	115.80	116.44
11	CH	118.10	117.14
14''	CH	119.60	119.85
2''	CH	121.50	120.01
2	CH	121.80	120.62
9''	CH	124.60	122.69
7a	C	124.80	125.69
10	CH	125.80	130.44
11a	C	127.60	132.50
4''	CH	133.10	133.58
9	C	134.20	134.54
7''	CH	134.40	135.10
6''	CH	139.00	137.43
5''	CH	140.20	139.51
8''	CH	141.10	140.96
3''	CH	144.40	141.40
17a''	C	145.60	145.14
16''	C	151.00	146.22
8	C	152.60	151.41

3	C	157.70	157.74
11’’	CO	158.70	159.15
13’’	C	158.70	159.45
1’’	CO	165.20	165.80
10’’	CO	166.10	166.55
7’	CO	169.60	170.27
12	CO	190.00	188.47
1	CO	196.00	195.52

(1) Only visible by feeding of [1,3-$^{13}C_2$] malonic acid.

Table S3

Bacterial strains used in this study.

Strain	Characteristics	Source/ Reference
E. coli		
DH5α	F', *supE44*, *lacU169*, (*Φ80lacZΔM15*), *ΔhsdR17*, *recA1*, *endA1*, *gyrA96*, *thi-1*, *relA1*	[2]
ET12567	F', *dam-13::Tn9* (Cml^R), *dcm-6*, *hsdM*, *hsdR*, *recF143, zij-202::Tn10* (Tet^R), *galK2*, *galT22*, *ara-14*, *lacY1*, *xyl-5*, *leuB6*, *thi-1*, *tonA31*, *rpsL136*, *hisG4*, *tsx-78*, *mtl-1*, *glnV44*	[3]
NR698	MC4100 (F- *araD139 Δ(argF-lac)U169 rpsL150 relA1 flbB5301 deoC1 ptsF25 rbsR*), *imp4213*	[4]
Rosetta(DE3) pLysS	F^-, *ompT, hsdSB*(*rB*$^-$ *mB*$^-$), *gal, dcm*, λ(DE3 [*lacI lacUV5-T7 gene1 ind1 sam7 nin5*]), pLysSRARE (Cml^R)	Novagen (Merck, Nottingham, UK)
Topo10	F^-, *mcrA*, *Δ(mrr-hsdRMSmcrBC)*, *Φ80lacZΔM15*, *ΔlacX74*, *recA1*, *araD139*, *Δ(ara-leu)7697*, *galU*, *galK*, *rpsL* (Str^R), *galE15 galK16 λ-endA1*, *nupG*	Invitrogen, Paisley, UK
Streptomyces		
S. antibioticus Tü6040	Natural producer of simocyclinone D8	[5]
S. coelicolor M1152	$SCP1^-$, $SCP2^-$ derivative of *S. coelicolor* with *Δact*, *Δred*, *Δcpk*, *Δcda*, *rpoB[C1298T]*	[6]
S. coelicolor M1152ex1	*S. coelicolor* M1152 with genomically integrated pIJ10480 (*ermEp*-simEx1*)	this study

Table S4

DNA constructs used in this study.

Plasmid	Characteristics	Source/ Reference
PAC-12I	pESAC13 with 85 kb *Bam*HI genomic region of the *sim* gene cluster from *S. antibioticus* Tü6040 (Kan^R, $Thio^R$)	this study
PAC-12IΔC7	PAC-12I with in frame deletion of *simC7*	this study
pESAC13	ΦC31, *attP-int, sacB, oriT*, P1 replicon (Kan^R, $Thio^R$) (derivative of pPAC-S1)	[7]
pET15b	Overexpression vector ($Carb^R$)	Novagen (Merck, Nottingham, UK)
pET15b-NB-C7	pET15b with *simC7* cloned between *Nde*I and *Bam*HI sites (N-terminal 6xHis) ($Carb^R$)	this study
pGM1190	*tsr*, *apr*, *oriT*, *to* terminator, *tipAp*, RBS, *fd* terminator (Apr^R)	[8]
pGM1190-NB-C7	pGM1190 with 855 bp *Nde*I-*Bam*HI fragment of *simC7* (*ptipA::simC7*)	this study
pGM1190-pB7-NB-C7	pGM1190 carrying *simC7* expressed from the *simB7* promoter (*pB7::simC7*)	this study
pIJ773	pBluescript KS (+) with the apramycin resistance gene *apr* and *oriT* from plasmid RP4, flanked by FRT sites (P1-FRT-*oriT*-*apr*-FRT-P2), (Apr^R)	[9]
pIJ773_del_oriT	pIJ773 without *oriT* (Apr^R)	this study
pIJ790	λ-RED (*gam*, *bet*, *exo*), *cat*, *araC*, $rep101^{ts}$ (Chl^R) (modified lambda-RED recombination plasmid pKD20)	[9]
pIJ10257	ΦBT1-based integrative vector containing the strong, constitutive *ermE** promoter (*ermEp**) (Hyg^R)	[10]
pIJ10480	pIJ10257 with *ermEp*-simEx1* (Hyg^R)	[11]
pJET1.2/blunt	*rep* (pMB1), *eco47IR*, P_{lacUV5}, T7 promoter, MCS, *bla* (Cml^R) (blunt end insertion site)	Fisher Scientific, Loughborough, UK
pR9406	self-transmissible helper plasmid derived from pUB307 ($Carb^R$)	A. Siddique and D. Figurski, unpublished

Table S5

DNA oligonucleotides used in this study. (underlined: start and stop codons of open reading frames; double underlined: introduced recognition sites for restrictions endonucleases).

Primer	Sequence
P1 (FRT-site)	5'-ATTCCGGGGATCCGTCGACC-3'
P2 (FRT-site)	5'-TGTAGGCTGGAGCTGCTTC-3'
right_wing_B2-F	5'-aacgagactgcccgtgtcttcacc-3'
right_wing_B2-R	5'-aggaacccgatcaccgacaggatg-3'
left_wing_B7-F	5'-tcaccgacgtcagcctgttcatcg-3'
left_wing_B7-R	5'-gttcatcgaccgtgtgcccttcttg-3'
PKS-C1A-F	5'-caactgccacctcgtactcac-3'
PKS-C1A-R	5'-gtacagcgtctcatccagcag-3'
check_del_C7-F	5'-ggtgctggtcctcctgcccgaggtgc-3'
check_del_C7-R	5'-tcccaccctgtacgccagaaaggtgc-3'
del_simC7-F	5'-ccggcttcgcccagacgggatgagaggacctgaacg***atg***ATTCCGGGGATCCGTCGACC-3'
del_simC7-R	5'-cgctgaacacccagggctgactgaacggcgctggga***cta***TGTAGGCTGGAGCTGCTTC-3'
simC7-NdeI-F	5'-GCA***cat*****ATG**aagattcttgtgaccggagc-3'
simC7-BamHI-R	5'-CAG**AGGCTT*****cta***aatgctctgagctgcc-3'
simC7-HindIII-R	5'-GCA**GGATCC*****cta***aatgctctgagctgcc-3'
pB7-NdeI-F	5'-tgaCATATGtcgttctgggcaggcatcag-3'
pB7-NdeI-R	5'-gcaCATATGtactccccattcagtggagg-3'
pGM1190-F	5'-ctgaggtcattactggaccg-3'
pGM1190-R	5'-tcgcaattcctttagttgttcc-3'
Apr-F	atgtcatcagcggtggagtgcaatg
Apr-R	tcagccaatcgactggcgag
T7-promoter-F	5'-taatacgactcactataggg-3'
T7-terminator-R	5'-gctagttattgctcagcgg-3'

Supplementary References

1. Holzenkampfer M, Walker M, Zeeck A, Schimana J, Fiedler HP. Simocyclinones, novel cytostatic angucyclinone antibiotics produced by *Streptomyces antibioticus* Tu 6040 II. Structure elucidation and biosynthesis. J Antibiot (Tokyo) 2002;55:301–7.

2. Hanahan D. Studies on transformation of *Escherichia coli* with plasmids. J Mol Biol 1983;166:557-80.

3. MacNeil DJ, Gewain KM, Ruby CL, Dezeny G, Gibbons PH, MacNeil T. Analysis of *Streptomyces avermitilis* genes required for avermectin biosynthesis utilizing a novel integration vector. Gene 1992;111:61-8.

4. Ruiz N, Falcone B, Kahne D, Silhavy TJ. Chemical conditionality: a genetic strategy to probe organelle assembly. Cell 2005;121:307-317.

5. Schimana J, Fiedler HP, Groth I, Sussmuth R, Beil W, Walker M, et al. Simocyclinones, novel cytostatic angucyclinone antibiotics produced by *Streptomyces antibioticus* Tu 6040. I. Taxonomy, fermentation, isolation and biological activities. J Antibiot (Tokyo) 2000;53:779–87.

6. Gomez-Escribano JP, Bibb MJ. Engineering *Streptomyces coelicolor* for heterologous expression of secondary metabolite gene clusters. Microbial Biotech 2011;4:207-215.

7. Sosio M, Giusino F, Cappellano C, Bossi E, Puglia AM, Donadio S. Artificial chromosomes for antibiotic-producing actinomycetes. Nat Biotechnol 2000;18:343-5.

8. Muth G, Nussbaumer B, Wohlleben W, Puhler A. A vector system with temperature-sensitive replication for gene disruption and mutational cloning in streptomycetes. Mol Gen Genet 1989;219:341-348.

9. Gust B, Challis GL, Fowler K, Kieser T, Chater KF. PCR-targeted *Streptomyces* gene replacement identifies a protein domain needed for biosynthesis of the sesquiterpene soil odor geosmin. Proc Natl Acad Sci U S A 2003;100:1541-1546.

10. Hong HJ, Hutchings MI, Hill LM, Buttner MJ. The role of the novel Fem protein VanK in vancomycin resistance in *Streptomyces coelicolor.* J Biol Chem 2005;280:13055-61.

11. Le TBK, Fiedler H-P, den Hengst CD, Ahn SK, Maxwell A, Buttner MJ. Coupling of the biosynthesis and export of the DNA gyrase inhibitor simocyclinone in *Streptomyces antibioticus*. Mol Microbiol 2009;72:1462-1474.
